# Predictive Factors and the Role of Conventionally Fractionated Radiation Therapy for Bone Metastasis from Renal Cell Carcinoma in the Era of Targeted Therapy

**DOI:** 10.3390/medicina60071049

**Published:** 2024-06-26

**Authors:** Hye Jin Kang, Myungsoo Kim, Yoo-Kang Kwak, So Jung Lee

**Affiliations:** Department of Radiation Oncology, Incheon St. Mary’s Hospital, College of Medicine, The Catholic University of Korea, Seoul 06591, Republic of Korea; mskim0710@gmail.com (M.K.); behappy1219@catholic.ac.kr (Y.-K.K.); sj_lee@catholic.ac.kr (S.J.L.)

**Keywords:** renal cell carcinoma, bone metastasis, radiation therapy, pain response, predictive factor

## Abstract

*Background and Objectives*: Despite rapid advances in targeted therapies for renal cell carcinoma (RCC), bone metastases remain a major problem that significantly increases morbidity and reduces patients’ quality of life. Conventional fractionated radiotherapy (CF-RT) is known to be an important local treatment option for bone metastases; however, bone metastases from RCC have traditionally been considered resistant to CF-RT. We aimed to investigate the effectiveness of CF-RT for symptomatic bone metastasis from RCC and identify the predictive factors associated with treatment outcomes in the targeted therapy era. *Materials and Methods*: Between January 2011 and December 2023, a total of 73 lesions in 50 patients treated with a palliative course of CF-RT for symptomatic bone metastasis from RCC were evaluated, and 62 lesions in 41 patients were included in this study. Forty-five lesions (72.6%) were treated using targeted therapy during CF-RT. The most common radiation dose fractionations were 30 gray (Gy) in 10 fractions (50%) and 39 Gy in 13 fractions (16.1%). *Results*: Pain relief was experienced in 51 of 62 lesions (82.3%), and the 12-month local control (LC) rate was 61.2%. Notably, 72.6% of the treatment course in this study was combined with targeted therapy. The 12-month LC rate was 74.8% in patients who received targeted therapy and only 10.9% in patients without targeted therapy (*p* < 0.001). Favorable Eastern Cooperative Oncology Group performance status (*p* = 0.026) and pain response (*p* < 0.001) were independent predictors of improved LC. Radiation dose escalation improved the LC in radiosensitive patients. A consistent treatment response was confirmed in patients with multiple treatment courses. *Conclusions*: CF-RT enhances pain relief and LC when combined with targeted therapy. Patients who responded well to initial treatment generally showed consistent responses to subsequent CF-RT for additional painful bone lesions. CF-RT could therefore be an excellent complementary local treatment modality for targeted therapy.

## 1. Introduction

Renal cell carcinoma (RCC) is the most common neoplasm of the kidney, accounting for approximately 2–3% of all new cancer diagnoses and deaths globally [1]. Although this condition occurs most commonly in North America and Western Europe, the incidence of RCC is rapidly increasing worldwide as more countries adopt Western lifestyles [2]. Since the bone is the second most common site of RCC metastasis after the lungs, bone metastasis from RCC is common [3]. Approximately 5–10% of patients have bone metastasis on initial presentation, and 20–35% of patients with RCC experience bone metastases during the disease course [4,5]. More than two-thirds of patients had multiple bone metastases [6]. Bone metastases from RCC are predominantly osteolytic lesions and are associated with skeletal-related events, including pain, impending fracture, and nerve compression, which can significantly increase morbidity and decrease patients’ quality of life [7]. Moreover, bone metastases have been reported to be an unfavorable prognostic factor for survival compared with other metastatic sites [8,9].

The introduction of targeted therapy has revolutionized the treatment strategies for metastatic RCC. Various targeted agents, such as tyrosine kinase inhibitors (TKIs) and monoclonal antibodies, are widely used as first- and second-line treatments for advanced RCC and have improved survival [10]. TKIs can extend the meantime to the progression of existing bone lesions and improve overall survival compared with the pre-TKI era [11,12]. Nivolumab, a monoclonal antibody against programmed cell death protein-1, improves survival in patients with bone metastases [13]. However, targeted therapy for bone metastases is only provided for palliative purposes. Achieving a complete response and preventing new bone metastases are challenging [12]; therefore, appropriate local treatment is often required for newly developed or worsening bone lesions during extended survival in the targeted therapy era. Conventionally fractionated radiation therapy (CF-RT) remains an important local treatment option for bone metastasis that can be used without restrictions, even in patients with either a poor performance status or disseminated metastatic disease (or both) with a limited life expectancy.

Here, we aimed to investigate the effectiveness of CF-RT in bone metastasis from RCC and identify the predictive factors associated with treatment outcomes to distinguish subgroups that may benefit clinically from palliative CF-RT in the targeted therapy era.

## 2. Materials and Methods

### 2.1. Patient Selection

Between January 2011 and December 2023, 73 lesions in 50 patients treated with a palliative course of CF-RT for symptomatic bone metastasis from RCC at our institution were retrospectively evaluated. Histological confirmation was performed to determine the primary tumor origin in all patients. All patients completed the planned RT. Patients were excluded if they had <1 month of follow-up or no imaging evaluation after the completion of treatment. Of the 50 patients, 11 lesions in 9 patients were excluded, and 62 lesions in 41 patients were included in the study. We collected data on the patients’ age, sex, histology, disease extent, treatment site, surgical intervention, pain severity, and evidence of disease progression. Lesion locations for bone metastasis were classified into four subtypes: long bone, vertebrae, pelvis, and others (skull, sternum, rib, and scapula).

This study was approved by the Institutional Review Board of Incheon St. Mary’s Hospital, the Catholic University of Korea (reference number: OC24RASI0052). All data were retrieved from medical reports and institutional medical records. The need for informed consent was waived owing to the study’s retrospective nature.

### 2.2. Treatment

Fourteen lesions (22.6%) underwent surgical intervention before CF-RT. Half of the lesions were located in the vertebrae and underwent debulking with decompression (laminectomy or corpectomy). The remaining lesions were four of long bones, two of scapula, and one of the pelvic bone. Curettage with cementation, internal fixation, and arthroplasty were performed in four, two, and one lesion, respectively.

Forty-five lesions (72.6%) were treated with targeted therapy during CF-RT. The administered agents were TKIs, monoclonal antibodies, and mammalian targets of rapamycin inhibitors, used in 24 (53.3%), 17 (37.8%), and 4 treatment courses (8.9%), respectively. Overall, 68.9% of lesions received first-line targeted therapy, while 31.1% received at least second-line therapy.

RT was administered to the bone sites for palliative pain. Prescribed treatment courses were at the attending physicians’ discretion based on clinical factors and physician preference. The researchers retrospectively collected the data. The radiation treatment courses were recorded as the total dose in gray (Gy) and the number of treatment fractions delivered. Radiation treatment courses ranged from 20 to 45 Gy in 5–15 fractionated treatments. The most used radiation dose fractionations were 30 Gy in 10 fractions (50%) and 39 Gy in 13 fractions (16.1%). The biologically effective dose (BED) was calculated for each treatment to account for the differences in dose and fractionation. BED was calculated using an α/β ratio of 7, as has been commonly used in previous studies [14,15]. The median BED was 42.9 (range: 31.4–64.3 Gy).

### 2.3. Response Evaluation and Statistical Analysis

Patients were assessed for clinical and radiographic responses following treatment. Using the visual analog scale, the severity of pain before CF-RT was evaluated based on patient reports from 0 to 10, with pain <8 classified as mild to moderate pain and pain ≥8 classified as severe pain. The initial pain response to irradiation was assessed at the 1-month follow-up after completion of CF-RT. Pain response was defined as a decrease of at least 2 points with no associated increase in the analgesic dose; non-response was defined as a decrease of within 1 point, stable or worse pain scale, or increased analgesic dose. Logistic regression tests were performed to identify the factors associated with initial pain response. Pain progression was defined as an increase in the pain scale score from the initial pain response. Local control (LC) was defined as the duration from the initiation of CF-RT to the date of either clinical or radiological progression, or the last follow-up visit for patients without disease progression. Radiological progression was defined as increased metastatic infiltration or soft tissue formation as detected by computed tomography and/or magnetic resonance imaging according to the Response Evaluation Criteria in Solid Tumors version 1.1 [16]. The Kaplan–Meier method was used to estimate the LC. Univariate analyses were performed using the Cox regression model to assess LC-related predictive factors. Potential prognostic factors (*p* < 0.100) in the univariate analyses were included in the multivariate analyses. Multivariate analyses were performed using the Cox proportional hazards model. All test results were two-sided. Statistical significance was set at *p* < 0.050. All statistical analyses were performed using the R software version 4.3.2. As each treatment site had a separate treatment course and pain assessment, each site was analyzed individually.

## 3. Results

### 3.1. Characteristics of the Patients and Lesion Treated

The characteristics of the patient and lesion treated are summarized in Table 1. Patient characteristics were categorized based on the time of first treatment. Most of the study cohort was male (73.2%), and clear cell subtype histology was the most common (85.4%). Internal organ metastases such as to the lung, liver, and brain were identified in 29 patients (70.7%). Of the total 41 patients, 30 had a single lesion, and 11 had multiple lesions: five patients had two lesions, four patients had three lesions, and the remaining two patients had four and six lesions, respectively. Among patients with multiple lesions, most lesions were treated at different times. Eastern Cooperative Oncology Group (ECOG) performance status and use of targeted therapy were obtained at the time of treatment. At the time of treatment, 14 lesions in 12 patients had an ECOG performance status ≥2. Pain severity was mild to moderate in 43 lesions (69.4%), and severe in 19 lesions (30.6%). Approximately half of the lesions were vertebral lesions.

### 3.2. Treatment Outcome

The median follow-up period was 10 months (range: 1–120 months). At the time of analysis, a total of 25 lesions (40.3%) exhibited local progression, and 29 patients (70.7%) had died. The median time to local progression was 20 months.

A total of 51 of 62 lesions (82.3%) among 33 patients experienced pain relief after treatment. A consistent pain response was confirmed in patients with multiple lesions. The nine patients who experienced pain relief during the initial treatment course also showed good responses when undergoing treatment for other lesions; however, the two patients who did not show pain control did not experience pain relief, even when other lesions were treated later. A favorable ECOG performance status (odds ratio [OR]: 0.09, *p* = 0.001) and the use of targeted therapy (OR: 12.44, *p* = 0.001) were associated with the initial pain response (Table 2). The median duration of pain relief was 11 months (range: 2–74 months), and 40 of 51 lesions had durable pain control at the final follow-up. The 6- and 12-month pain control rates were 95.0% and 82.7%, respectively.

**Figure 1:**
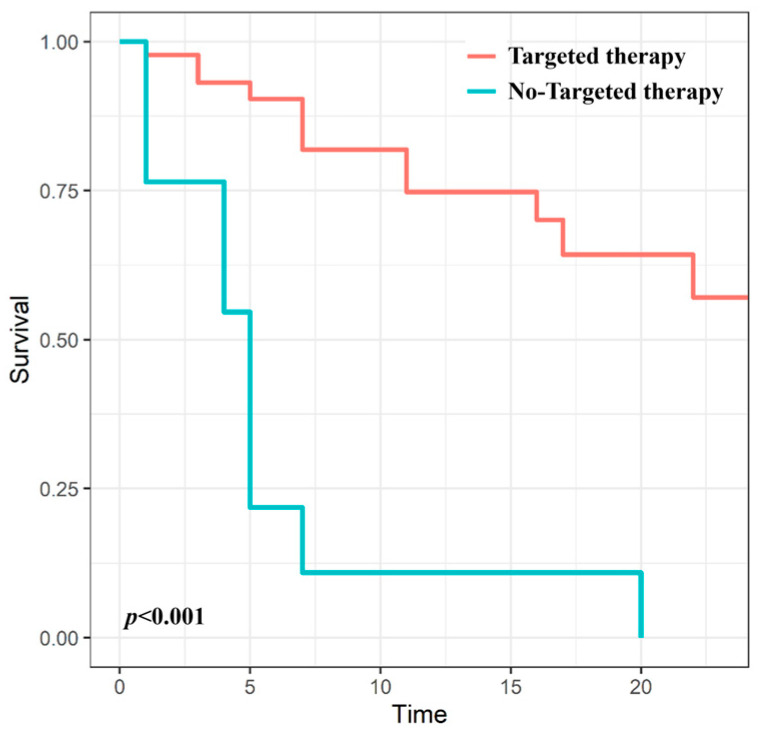
Kaplan–Meier curves for local control according to targeted therapy use.

The 6- and 12-month LC rates were 75.7% and 61.2%, respectively. Regarding the use of targeted therapy, the 12-month LC rate was 74.8% in patients with targeted therapy and only 10.9% in patients without targeted therapy (*p* < 0.001) (Figure 1).

### 3.3. Predictive Factors for LC

**Figure 2:**
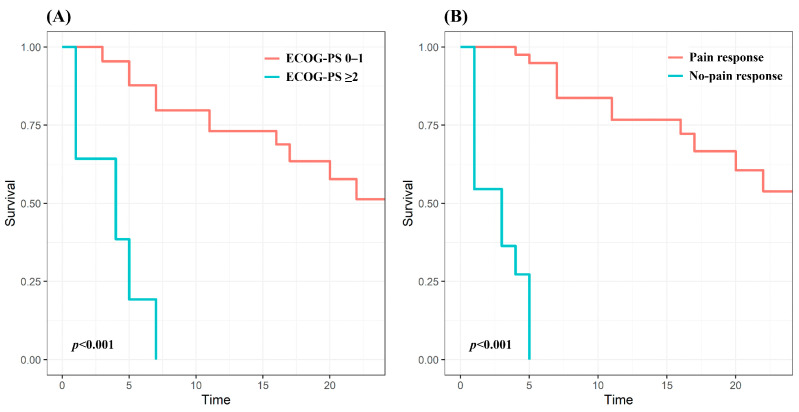
Kaplan–Meier curves for local control according to (**A**) ECOG performance status and (**B**) pain response. ECOG-PS, Eastern Cooperative Oncology Group performance status.

To evaluate predictive factors associated with LC, the following parameters were analyzed: age (<60 vs. ≥60 years), sex (male vs. female), ECOG performance status at the time of treatment (0–1 vs. ≥2), histology (clear cell subtype vs. other), nephrectomy (yes vs. no), visceral metastases (yes vs. no), concurrent targeted therapy (yes vs. no), severity of pain (mild to moderate vs. severe), surgical intervention (yes vs. no), soft tissue formation (yes vs. no), pain response (yes vs. no), and irradiation dose (BED, ≤42.9 Gy vs. >42.9 Gy). The significant factors from the univariate and multivariate analyses are presented in Table 3. Targeted therapy only had a statistically significant association with LC in the univariate analysis, losing significance in the multivariate analysis. A favorable ECOG performance status (hazard ratio [HR]: 2.01, *p* = 0.026) and pain response (HR: 0.03, *p* < 0.001) remained independent predictors of improved LC. The 12-month LC rates were 73.1% in patients with a favorable performance status, and 76.7% in patients with pain response; the 12-month LC rate of patients with a poor performance status or pain not relieved by CF-RT was 0% (Figure 2).

**Figure 3:**
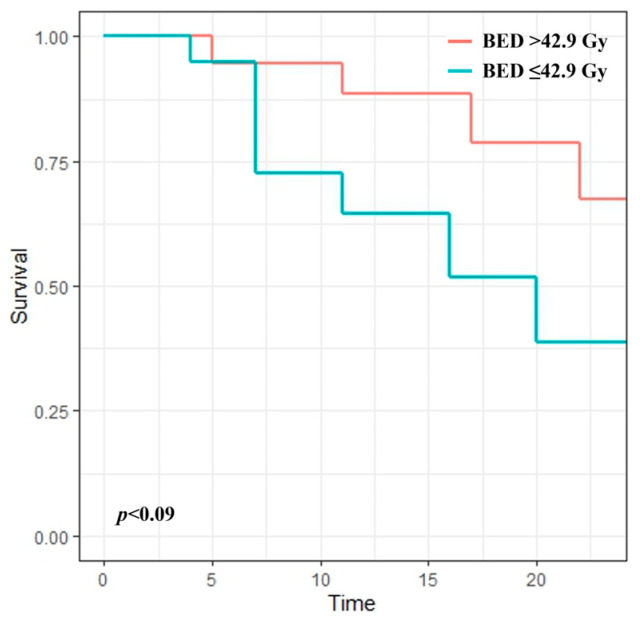
Kaplan–Meier curves for local control according to radiation dose in the subgroup with a pain response.

The irradiation dose was not associated with LC in the entire cohort; however, irradiation dose was the only independent predictor of LC in the subgroup with a pain response with borderline significance (HR: 0.93, *p* = 0.054). The 12-month LC rate of patients receiving a BED > 42.9 Gy was 88.4%, whereas that of patients receiving a BED ≤ 42.9 Gy was 64.6% (Figure 3).

## 4. Discussion

Rapid advances in targeted therapies for RCC have improved overall survival but have a limited effect on preventing the progression of existing bone metastasis or the occurrence of new bone metastasis [10,12]. Therefore, bone metastases still remain a major problem that causes significant morbidity and reduces patients’ quality of life; appropriate local treatment is more often required for newly developed or worsening bone lesions during extended survival in the targeted therapy era [7]. Although RT is a representative local treatment option for cancer, bone metastases from RCC were traditionally considered resistant to RT [17]. An early phase II study to evaluate the efficacy of CF-RT in bone metastases from RCC demonstrated a significant response rate of 83%; however, results were limited with a median duration of response after treatment of 3 months [18]. Advances in technology have led to the increasing utilization of stereotactic body RT (SBRT) to overcome radioresistance in the treatment of bone metastases from RCC. In a retrospective study comparing the effects of CF-RT and SBRT on bone metastasis from RCC, 12-month LC rates were significantly higher for SBRT (74.9% vs. 39.9%; *p* < 0.001) [14]. However, SBRT may only be considered in patients with limited conditions, such as those with single to oligometastases and small-sized tumors [19]. SBRT is not possible if the metastases are systemically disseminated or the size of the target is too large. Therefore, CF-RT remains an important treatment option that can be used for the local treatment of bone metastasis without restrictions [20].

We evaluated the effect of CF-RT on pain relief and LC in painful bone metastases from RCC in the era of targeted therapy. This study only included patients after the introduction of targeted therapy, and 72.6% of the treatment course in this study was combined with targeted therapy. When combined with targeted therapy, a significant improvement in pain response and LC was demonstrated. The pain response of patients treated with targeted therapy was 93.3%; however, a pain response was only identified in half of patients without targeted therapy. The 12-month LC rate was 74.8% in patients with targeted therapy and only 10.9% in patients without targeted therapy (*p* < 0.001). Targeted therapy only had a statistically significant association with LC in the univariate analysis and lost significance in the multivariate analysis. Rather than demonstrating a truly low significance, this finding may reflect a lack of statistical power due to the small number of patients in the non-targeted therapy group. Published data on the effectiveness of CF-RT in combination with targeted therapies are currently limited. Ansari et al. conducted a retrospective study in which patients with metastatic RCC were treated with CF-RT when progression occurred while using nivolumab. Approximately 70% of the radiation treatment sites were metastatic bone lesions, and 72% of lesions showed more than partial response. Therefore, the addition of CF-RT appeared to initiate a treatment response and prolong the duration of nivolumab treatment [21]. Recently reported studies for bone metastasis from RCC are consistent with our improved treatment outcomes compared to early studies [22,23]; however, regarding the potential synergistic effect of combined targeted therapy, heterogeneous results were observed. Makita et al. evaluated the palliative CF-RT effect on radioresistant carcinoma, including RCC; the 12-month LC rate was 62%, and the administration of TKIs was associated with improved LC in multivariate analysis (HR 2.19, *p* = 0.010) [22]. Lee et al. reported the outcome of spinal metastases from RCC treated with CF-RT [23]; the 12-month LC rate was 87.5%, and the use of TKIs was the only independent predictor of improved overall survival (HR 0.47, *p* = 0.050).

Furthermore, we identified patients who would receive the greatest clinical benefit from palliative CF-RT. Pain response and LC correlated with performance status. Many prior studies have reported that performance status was a major predictive factor for treatment outcomes [23,24,25]; still, there is no clear explanation as to why performance status affects treatment outcomes. Velden et al. explained that decreasing performance status reflects the decline in physiological and immunological functions necessary to produce analgesic effects after irradiation and created a response prediction model including performance status [25]. A poor performance status may itself reflect the overall disease progression status and make it difficult to tolerate targeted therapy, ultimately leading to short survival. LC was also closely associated with pain response, as well as performance status. All patients whose pain was not relieved by CF-RT showed progression within 6 months. Ganju et al. found that pain response was the only predictor of LC, and the median time to radiographic progression was significantly improved in the responder group (22.8 months vs. 1.5 months, *p* < 0.001) [15]. Initial pain response may, therefore, serve as a surrogate for tumor response or radiosensitivity.

Several studies have evaluated the relationship between radiation dose and LC, showing heterogeneous results. Makita et al. found that an elevated CF-RT dose has a significant impact on the 12-month LC rate (55% vs. 84%, *p* = 0.02) [22]. However, elevated doses did not show an additional LC benefit in other studies [15,26,27]. In our study, no significant association was found between radiation dose and LC in the entire cohort. However, irradiation dose was an independent predictor of LC in the subgroup with a pain response. The 12-month LC rate of patients receiving a BED > 42.9 Gy was 88.4%, whereas that of patients receiving a BED ≤ 42.9 Gy was 64.6%. This finding indicates that dose escalation seems to prolong the progression in radiosensitive patients.

Additionally, a quarter of patients included in this study had ≥2 treated lesions. These patients had a pain response to additional treatment consistent with their initial response to the first course of treatment; therefore, the response to the first course of treatment may predict the treatment response to subsequent additional treatment courses. Although we were unable to find data evaluating the concordance of treatment response to palliative CF-RT in multiple bone metastasis, prior studies have demonstrated that patients who experience pain relief during initial treatment are more likely to achieve effective pain relief with re-irradiation [28,29]. This may help determine the direction of further treatment and provide a viable option for additional bone lesions.

This study had some limitations in addition to its retrospective nature. First, the dataset included a heterogeneous population of patients. The disease extent varied, and patients received various types of targeted therapy and different schedules of CF-RT. This might have affected treatment outcomes in this study. Furthermore, this study included a relatively small number of patients and a short follow-up period; the patient groups used in the subgroup analysis were also of a small size. Further studies are thus required to analyze the data of larger numbers of patients with longer follow-up periods. Finally, patient symptoms and pain response might not have been fully documented in the medical records and, therefore, may have been underestimated. Despite these limitations, this study identified the effect of CF-RT in bone metastases from RCC, as well as the predictive factors associated with treatment outcomes, the benefit of an elevated radiation dose, and subpopulations of patients who can benefit from CF-RT.

## 5. Conclusions

Advances in targeted therapies for RCC have improved overall survival but have had a limited impact on bone metastasis. This has led to a rise in the number of cases requiring local treatment for bone metastases. Traditionally, bone metastases from RCC were considered resistant to CF-RT. However, we identified that CF-RT enhances pain relief and LC when combined with targeted therapy. Furthermore, while pain relief is an important treatment goal in itself, it may also serve as an early predictor of LC or radiosensitivity. Patients who responded well to initial treatment generally showed consistent responses to subsequent CF-RT for additional painful bone lesions, making it a viable option for ongoing management. Lastly, dose escalation may extend the duration of LC in radiosensitive patients. Providing elevated radiation doses may offer additional benefits, particularly for patients with longer life expectancy. Based on these findings, it was confirmed that CF-RT can be an excellent complementary treatment modality to targeted therapy as a local treatment.

## Figures and Tables

**Table 1:** Characteristics of the patient, lesion, and treatment.

Characteristics	No. (%)
Patient characteristics	
Number of patients	41 (100)
Age (years)	
<60	19 (46.3)
≥60	22 (53.7)
Sex	
Male	30 (73.2)
Female	11 (26.8)
Histology	
Clear cell subtype	35 (85.4)
Other	6 (14.6)
Nephrectomy	
Yes	15 (36.6)
No	26 (63.4)
Internal organs metastases	
Yes	29 (70.7)
No	12 (29.3)
Number of bone metastatic lesions	
Single	19 (46.3)
≤3	9 (22.0)
>3	13 (31.7)
Lesion and treatment characteristics	
Number of treatment lesions	62 (100)
ECOG-PS at the time of treatment	
0–1	48 (77.4)
≥2	14 (22.6)
Type of targeted therapy	
TKI	24 (53.3%)
Monoclonal antibody	17 (37.8%)
mTOR inhibitor	4 (8.9%)
No	17 (27.4)
Chronology of treatment lesions	
Synchronous	32 (51.6)
Metachronous	30 (48.4)
Surgical intervention	
Yes	14 (22.6)
No	48 (77.4)
Severity of pain	
Mild to moderate	43 (69.4)
Severe	19 (30.6)
Lesion location	
Long bone	7 (11.3)
Vertebra	32 (51.6)
Pelvis	7 (11.3)
Other	16 (25.8)
Soft tissue mass formation	
Present	27 (43.5)
Absent	35 (56.5)
Radiation therapy course	
30 Gy in 10 fractions	31 (50.0)
39 Gy in 13 fractions	10 (16.1)
40 Gy in 10 fractions	8 (12.9)
36 Gy in 12 fractions	6 (9.7)
35 Gy in 10 fractions	3 (4.9)
20 Gy in 5 fractions	2 (3.2)
45 Gy in 15 fractions	1 (1.6)
30 Gy in 5 fractions	1 (1.6)
Median BED (range)	42.9 (31.4–64.3)

ECOG-PS, Eastern Cooperative Oncology Group performance status; TKI, tyrosine kinase inhibitor; mTOR, mammalian targets of rapamycin; Gy, gray; BED, biologically effective dose.

**Table 2:** Initial pain response rate according to clinical factors.

Characteristics	Pain Response No. (%)	*p*-Value
Age		0.183
<60	30/34 (88.2)	
≥60	21/28 (75.0)	
Histology		0.994
Clear cell subtype	43/54 (79.6)	
Other	8/8 (100.0)	
ECOG-PS		0.001 *
0–1	44/48 (91.7)	
≥2	7/14 (50.0)	
Surgical intervention		0.992
Yes	14/14 (100.0)	
No	37/48 (77.1)	
Targeted therapy		0.001 *
Yes	42/45 (93.3)	
No	9/17 (52.9)	
Severity of pain		0.247
Mild to moderate	37/43 (86.0)	
Severe	14/19 (73.7)	
Soft tissue formation		0.888
Present	29/35 (82.9)	
Absent	22/27 (81.5)	
Radiation dose		0.923
BED ≤ 42.9 Gy	27/33 (81.8)	
BED > 42.9 Gy	24/29 (82.8)	

ECOG-PS, Eastern Cooperative Oncology Group performance status; BED, biologically effective dose; Gy, gray. * Statistically significant.

**Table 3:** Univariate and multivariate analyses for local control.

Variable	Univariate Analysis		Multivariate Analysis	
	HR (95% CI)	*p*-Value	HR (95% CI)	*p*-Value
Sex (male vs. female)	0.32 (0.14–0.75)	0.009		
ECOG-PS (0–1 vs. ≥2)	3.86 (2.29–6.60)	<0.001	2.01 (1.08–3.71)	0.026
Targeted therapy (yes vs. no)	0.12 (0.05–0.29)	<0.001		
Surgical intervention (yes vs. no)	0.29 (0.10–0.88)	0.029		
Pain response (yes vs. no)	0.02 (0.00–0.08)	<0.001	0.03 (0.01–0.16)	<0.001

ECOG-PS, Eastern Cooperative Oncology Group performance status; HR, hazard ratio; CI, confidence interval.

## Data Availability

Data are available upon reasonable request.

## References

[1] Graves A., Hessamodini H., Wong G., Lim W.H. (2013). Metastatic renal cell carcinoma: Update on epidemiology, genetics, and therapeutic modalities. Immunotargets Ther..

[2] Padala S.A., Barsouk A., Thandra K.C., Saginala K., Mohammed A., Vakiti A., Rawla P., Barsouk A. (2020). Epidemiology of Renal Cell Carcinoma. World J. Oncol..

[3] Zekri J., Ahmed N., Coleman R.E., Hancock B.W. (2001). The skeletal metastatic complications of renal cell carcinoma. Int. J. Oncol..

[4] Guo Q., Zhang C., Guo X., Tao F., Xu Y., Feng G., Han X., Ren Z., Zhang H., Zhang P. (2018). Incidence of bone metastasis and factors contributing to its development and prognosis in newly diagnosed renal cell carcinoma: A population-based study. Cancer Manag. Res..

[5] Gupta K., Miller J.D., Li J.Z., Russell M.W., Charbonneau C. (2008). Epidemiologic and socioeconomic burden of metastatic renal cell carcinoma (mRCC): A literature review. Cancer Treat. Rev..

[6] Santini D., Procopio G., Porta C., Ibrahim T., Barni S., Mazzara C., Fontana A., Berruti A., Berardi R., Vincenzi B. (2013). Natural history of malignant bone disease in renal cancer: Final results of an Italian bone metastasis survey. PLoS ONE.

[7] Beuselinck B., Oudard S., Rixe O., Wolter P., Blesius A., Ayllon J., Elaidi R., Schöffski P., Barrascout E., Morel A. (2011). Negative impact of bone metastasis on outcome in clear-cell renal cell carcinoma treated with sunitinib. Ann. Oncol..

[8] Motzer R.J., Escudier B., Bukowski R., Rini B.I., Hutson T.E., Barrios C.H., Lin X., Fly K., Matczak E., Gore M.E. (2013). Prognostic factors for survival in 1059 patients treated with sunitinib for metastatic renal cell carcinoma. Br. J. Cancer.

[9] McKay R.R., Kroeger N., Xie W., Lee J.L., Knox J.J., Bjarnason G.A., MacKenzie M.J., Wood L., Srinivas S., Vaishampayan U.N. (2014). Impact of bone and liver metastases on patients with renal cell carcinoma treated with targeted therapy. Eur. Urol..

[10] National Comprehensive Cancer Network Kidney Cancer (Version 3.2024). https://www.nccn.org/professionals/physician_gls/pdf/kidney.pdf.

[11] Zołnierek J., Nurzyński P., Langiewicz P., Oborska S., Waśko-Grabowska A., Kuszatal E., Obrocka B., Szczylik C. (2010). Efficacy of targeted therapy in patients with renal cell carcinoma with pre-existing or new bone metastases. J. Cancer Res. Clin. Oncol..

[12] Kalra S., Verma J., Atkinson B.J., Matin S.F., Wood C.G., Karam J.A., Lin S.H., Satcher R.L., Tamboli P., Sircar K. (2017). Outcomes of Patients with Metastatic Renal Cell Carcinoma and Bone Metastases in the Targeted Therapy Era. Clin. Genitourin. Cancer.

[13] Motzer R.J., Escudier B., McDermott D.F., George S., Hammers H.J., Srinivas S., Tykodi S.S., Sosman J.A., Procopio G., Plimack E.R. (2015). Nivolumab versus Everolimus in Advanced Renal-Cell Carcinoma. N. Engl. J. Med..

[14] Amini A., Altoos B., Bourlon M.T., Bedrick E., Bhatia S., Kessler E.R., Flaig T.W., Fisher C.M., Kavanagh B.D., Lam E.T. (2015). Local control rates of metastatic renal cell carcinoma (RCC) to the bone using stereotactic body radiation therapy: Is RCC truly radioresistant?. Pract. Radiat. Oncol..

[15] Ganju R.G., TenNapel M., Mahan N., Zahra A., Shen X. (2018). The Efficacy of Conventionally Fractionated Radiation in the Management of Osseous Metastases from Metastatic Renal Cell Carcinoma. J. Oncol..

[16] Eisenhauer E.A., Therasse P., Bogaerts J., Schwartz L.H., Sargent D., Ford R., Dancey J., Arbuck S., Gwyther S., Mooney M. (2009). New response evaluation criteria in solid tumours: Revised RECIST guideline (version 1.1). Eur. J. Cancer.

[17] Wei Q., He H., Lv L., Xu X., Sun W. (2020). The promising role of radiotherapy in the treatment of advanced or metastatic renal cell carcinoma: A narrative review. Transl. Androl. Urol..

[18] Lee J., Hodgson D., Chow E., Bezjak A., Catton P., Tsuji D., O’Brien M., Danjoux C., Hayter C., Warde P. (2005). A phase II trial of palliative radiotherapy for metastatic renal cell carcinoma. Cancer.

[19] Lo S.S., Fakiris A.J., Chang E.L., Mayr N.A., Wang J.Z., Papiez L., Teh B.S., McGarry R.C., Cardenes H.R., Timmerman R.D. (2010). Stereotactic body radiation therapy: A novel treatment modality. Nat. Rev. Clin. Oncol..

[20] De Felice F., Piccioli A., Musio D., Tombolini V. (2017). The role of radiation therapy in bone metastases management. Oncotarget.

[21] Ansari J., Farrag A., Ali A., Abdelgelil M., Murshid E., Alhamad A., Ali M., Ansari H., Hussain S., Glaholm J. (2021). Concurrent use of nivolumab and radiotherapy for patients with metastatic non-small cell lung cancer and renal cell carcinoma with oligometastatic disease progression on nivolumab. Mol. Clin. Oncol..

[22] Makita K., Hamamoto Y., Kanzaki H., Nagasaki K., Takata N., Tsuruoka S., Uwatsu K., Kido T. (2023). Factors affecting local control of bone metastases from radioresistant tumors treated with palliative external beam radiotherapy. Discov. Oncol..

[23] Lee C.C., Tey J.C.S., Cheo T., Lee C.H., Wong A., Kumar N., Vellayappan B. (2020). Outcomes of patients with spinal metastases from renal cell carcinoma treated with conventionally-fractionated external beam radiation therapy. Medicine.

[24] Ryu S., Deshmukh S., Timmerman R.D., Movsas B., Gerszten P., Yin F.F., Dicker A., Abraham C.D., Zhong J., Shiao S.L. (2023). Stereotactic Radiosurgery vs Conventional Radiotherapy for Localized Vertebral Metastases of the Spine: Phase 3 Results of NRG Oncology/RTOG 0631 Randomized Clinical Trial. JAMA Oncol..

[25] van der Velden J.M., Peters M., Verlaan J.J., Versteeg A.L., Zhang L., Tsao M., Danjoux C., Barnes E., van Vulpen M., Chow E. (2017). Development and Internal Validation of a Clinical Risk Score to Predict Pain Response after Palliative Radiation Therapy in Patients with Bone Metastases. Int. J. Radiat. Oncol. Biol. Phys..

[26] Wilson D., Hiller L., Gray L., Grainger M., Stirling A., James N. (2003). The effect of biological effective dose on time to symptom progression in metastatic renal cell carcinoma. Clin. Oncol..

[27] Schlampp I., Lang H., Förster R., Wolf R., Bostel T., Bruckner T., Debus J., Rief H. (2015). Stability of spinal bone metastases and survival analysis in renal cancer after radiotherapy. Tumori.

[28] Tsukamoto S., Kido A., Tanaka Y., Facchini G., Peta G., Rossi G., Mavrogenis A.F. (2021). Current Overview of Treatment for Metastatic Bone Disease. Curr. Oncol..

[29] Chow E., van der Linden Y.M., Roos D., Hartsell W.F., Hoskin P., Wu J.S., Brundage M.D., Nabid A., Tissing-Tan C.J., Oei B. (2014). Single versus multiple fractions of repeat radiation for painful bone metastases: A randomised, controlled, non-inferiority trial. Lancet Oncol..

